# Investigations into the Role of Metabolism in the Inflammatory Response of BV2 Microglial Cells

**DOI:** 10.3390/antiox10010109

**Published:** 2021-01-14

**Authors:** Pamela Maher

**Affiliations:** Cellular Neurobiology Laboratory, Salk Institute for Biological Studies, La Jolla, CA 92037, USA; pmaher@salk.edu

**Keywords:** glycolysis, oxidative phosphorylation, reactive oxygen species, mitochondria, cytokines, diabetes

## Abstract

Although the hallmarks of Alzheimer’s disease (AD) are amyloid beta plaques and neurofibrillary tangles, there is growing evidence that neuroinflammation, mitochondrial dysfunction and oxidative stress play important roles in disease development and progression. A major risk factor for the development of AD is diabetes, which is also characterized by oxidative stress and mitochondrial dysfunction along with chronic, low-grade inflammation. Increasing evidence indicates that in immune cells, the induction of a pro-inflammatory phenotype is associated with a shift from oxidative phosphorylation (OXPHOS) to glycolysis. However, whether hyperglycemia also contributes to this shift is not clear. Several different approaches including culturing BV2 microglial cells in different carbon sources, using enzyme inhibitors and knocking down key pathway elements were used in conjunction with bacterial lipopolysaccharide (LPS) activation to address this question. The results indicate that while high glucose favors NO production, pro-inflammatory cytokine production is highest in the presence of carbon sources that drive OXPHOS. In addition, among the carbon sources that drive OXPHOS, glutamine is a very potent inducer of IL6 production. This effect is dampened in the presence of glucose. Together, these results may provide new prospects for the therapeutic manipulation of neuroinflammation in the context of diabetes and AD.

## 1. Introduction

Alzheimer’s disease (AD) is the most common age-associated disease but there are no treatments that can prevent, delay, slow or stop its progression. While AD is characterized by both the abnormal aggregation of hyperphosphorylated tau protein which gives rise to neurofibrillary tangles and amyloid β (Aβ) accumulation in extracellular plaques [1], neuroinflammation and oxidative stress are thought to be important contributors to AD pathology and neurodegeneration [2,3,4,5]. Indeed, there is now substantial genetic and experimental evidence indicating that the enhanced activation of glial cells (both microglia and astrocytes) in the brain results in inflammatory consequences that are important for the progression of AD and may even help to drive AD pathology independently of Aβ deposition or tau aggregation [4]. However, the causes of this neuroinflammation remain unclear. 

A number of epidemiological studies have suggested a link between diabetes and AD [6,7], with an increased risk ranging from 1.5- to 2-fold. Moreover, this link appears to be strongly associated with the chronic hyperglycemia that is the hallmark of diabetes and is thought to be the major cause of diabetic complications [8,9]. Furthermore, hyperglycemia leads to oxidative stress, which is due to the accumulation of reactive oxygen species (ROS) that are generated at least partly as a result of mitochondrial dysfunction and are central to the pathological consequences of diabetes [10,11,12]. Together, these observations suggest that hyperglycemia could play a critical role in the exacerbation of neuroinflammation and oxidative stress that contribute to the development and progression of AD. 

Increasing evidence suggests that metabolic reprogramming plays a critical role in immune cell activation [13,14]. In macrophages and dendritic cells, the induction of a pro-inflammatory phenotype is associated with a shift from oxidative phosphorylation (OXPHOS) to glycolysis [15,16]. The available evidence suggests that this also occurs in microglia [17,18,19,20,21]. However, whether this switch can be enhanced by hyperglycemia and the role that it plays in the induction of the hallmarks of a pro-inflammatory phenotype in microglia, including increased NO production and pro-inflammatory cytokine synthesis, is not entirely clear.

In order to better understand how both high glucose and mitochondrial dysfunction could contribute to neuroinflammation in the context of AD, I decided to focus on microglial cells, the resident immune cells of the brain. For these studies, I used the mouse BV2 microglial cell line for several reasons. First, a large number of cells were needed for these studies which was much more than could be efficiently generated using primary cultures. Second, the BV2 cell line has been very well characterized, is considered an excellent in vitro microglial cell model [22] and has been used to demonstrate a metabolic shift in response to a pro-inflammatory stimulus such as bacterial lipopolysaccharide (LPS) [17,19]. Third, using a cell line rather than primary cells greatly reduces experiment-to-experiment variability. Importantly, there is growing evidence that microglia in the aging brain undergo significant changes that result in a chronically activated phenotype [23,24]. Whether additional stresses such as those that occur in the context of diabetes could further exacerbate this age-related pro-inflammatory phenotype and thereby contribute to the development of AD is not clear. In the experiments described herein, the BV2 cells were exposed to both LPS and a variety of different treatments designed to modulate glucose levels, mitochondrial function and oxidative stress in order to determine whether these treatments could enhance an ongoing pro-inflammatory response. The results show that both high glucose and mitochondrial dysfunction modulate the pro-inflammatory phenotype of microglial cells in distinct ways that could be detrimental to brain function. 

## 2. Materials and Methods 

### 2.1. Materials

Bacterial lipopolysaccharide (LPS), iodoacetic acid (IAA), 2-deoxyglucose (2DG), indomethacin, 2-thenoyltrifluoroacetone (TTFA), oligomycin, rotenone, antimycin A and myxothiazol were from Sigma Aldrich (St. Louis, MO, USA). GKT137831, zileuton, PD146176, idebenone, piericidin A, trifluoromethoxy carbonylcyanide phenylhydroazone (FCCP), S-ethyl isothiourea (SEITU) and 1400W were from Cayman Chemical (Ann Arbor, MI, USA). GSK2795039 was from MedChem Express (Monmouth Junction, NJ, USA). All other chemicals were from Sigma Aldrich.

### 2.2. BV2 Cell Culture and Treatment

Mouse BV2 microglial cells (obtained from Dr. Grace Y. Sun (University of Missouri at Columbia, MO, USA) were routinely grown in low-glucose (5 mM) DMEM (Thermo Fisher/Gibco, Waltham, MA, USA) supplemented with 10% fetal calf serum (FCS) (Gibco) and antibiotics (Gibco) at 10% CO_2_. For all of the assays unless otherwise noted, the cells were plated at 5 × 10^5^ cells in 35 mm tissue culture dishes. After overnight growth, the cells were transferred to high-glucose (25 mM) DMEM supplemented with 10% FCS and treated with 25 ng/mL LPS alone or in the presence of the different compounds which were added 30 min before the treatment with LPS. After 24 h, the medium was removed, spun briefly to remove floating cells and 100 µL was assayed for nitrite, the stable oxidative end product of NO and therefore a measure of its production, using 100 µL of the Griess Reagent (Sigma Aldrich) in a 96 well plate. After incubation for 10 min at room temperature, the absorbance at 550 nm was read on a Molecular Devices microplate reader. The remaining culture supernatants were stored at −20 °C until used to determine their levels of IL6 and TNFα using ELISAs (R&D Systems, Minneapolis, MN, USA) according to the manufacturer’s instructions. In order to control for differential effects of the various treatments on cell survival, the cells left in the dishes after removal of the culture supernatants were assayed for viability using the MTT assay as described previously [25]. NO or cytokine production was normalized to the MTT assay results. Because the absolute levels of NO or cytokine production varied somewhat from experiment to experiment, in order to compare the results from different experiments, the levels were normalized to the level seen with LPS alone. Absolute levels of nitrite ranged from 8 to 12 µM, IL6 from 400 to 700 ng/mL and TNFα from 300 to 500 ng/mL in the different experiments. In all cases, the absolute levels of nitrite, IL6 and TNFα in the untreated BV2 cells were minimal.

### 2.3. Manipulation of Carbon Source

To alter the carbon source used by the cells for metabolism, the approach described by Lanning et al. [26] was used. Briefly, 5 × 10^5^ cells BV2 cells were grown overnight in 35 mm dishes. The cells were rinsed twice in phosphate-buffered saline (PBS) and then the cells were put into DMEM containing the desired carbon source. To do this, phenol red free DMEM without glucose, pyruvate or glutamine (Gibco) with 10% dialyzed FCS and antibiotics was used as a base and then glucose, pyruvate or glutamine was added from frozen stocks as needed to a final concentration of 10 mM each. The cells were then treated with LPS and analyzed as described above for NO and cytokine production.

### 2.4. Intracellular ATP Assay

Intracellular ATP was determined using lysates from cells exposed to the different carbon sources in the absence or presence of 10 µM IAA or 10 µM rotenone. Briefly, 1 × 10^6^ BV2 cells were plated in 60 mm dishes. After 24 h of culture, the cells were rinsed and the medium was exchanged with medium containing each of the different carbon sources and IAA or rotenone was added as indicated to block glycolysis or mitochondrial respiration, respectively. After 4 h, the cells were washed twice in cold PBS without calcium or magnesium and then scraped into lysis buffer containing 50 mm HEPES, pH 7.4, 150 mm NaCl, 50 mm NaF, 1.5 mm MgCl_2_, 1 mm EDTA, 10 mm sodium pyrophosphate, 1 mm Na_3_VO_4_ and 1% Triton X-100. Lysates were incubated at 4 °C for 30 min, then cleared by centrifugation at 14,000× *g* for 10 min. ATP levels were determined using a chemiluminescent kit from ThermoFisher/Invitrogen and normalized to total cellular protein as determined using the bicinchoninic acid (BCA) assay (ThermoFisher/Pierce). 

### 2.5. Transfection

For siRNA transfection, BV2 cells were plated in 60 mm dishes at 1 × 10^6^ cells/dish and 20 pmol IF1 siRNA (#sc-141374) or iNOS siRNA (#sc-36092) from Santa Cruz Biotechnology (Dallas, TX, USA) or control siRNA (#1027280) from Qiagen (Germantown, MD, USA), were used along with RNAi max (ThermoFisher/Invitrogen) according to the manufacturer’s instructions. The average reduction in IF1 levels was 85% and in iNOS levels was 90%.

### 2.6. Western Blotting

*Sample Preparation:* For Western blotting, 5 × 10^5^ BV2 cells per 35 mm dish were grown for 24 h prior to the indicated treatments. Total protein extracts were prepared by rinsing the cells twice with ice-cold PBS without calcium or magnesium, scraping the cells into lysis buffer containing 1× protease inhibitor cocktail and 1× phosphatase inhibitor cocktail and incubating on ice for 30 min. Extracts were sonicated and cleared by centrifugation. The supernatants were stored at −70 °C until analysis. Protein concentrations were quantified by the BCA assay and adjusted to equal concentrations. Thereafter, 5× Western blot sample buffer (74 mM Tris-HCl, pH 8.0, 6.25% SDS, 10% β-mercaptoethanol, 20% glycerol) was added to a final concentration of 2.5× and samples were boiled for 5 min. 

*Western blotting:* For SDS-PAGE, equal amounts of cellular protein, typically 10–20 µg per lane, were used. All samples were separated using 4–12% Criterion XT Precast Bis-Tris Gels (Biorad, Hercules, CA, USA). Proteins were transferred to nitrocellulose membranes and the quality of protein measurement, electrophoresis and transfer determined by staining with Ponceau S. Membranes were blocked with 5% skim milk in TBS-T (20 mM Tris buffer pH 7.5, 0.5 M NaCl, 0.1% Tween 20) for 1 h at room temperature and incubated overnight at 4 °C in the primary antibody diluted in 5% BSA in TBS/0.05% Tween 20. The primary antibodies used were: rabbit anti-IF1 antibody from Santa Cruz Biotechnology (#sc134962, 1/1000) and mouse anti-iNOS antibody (#610431, 1/1000) from BD Transduction Labs (San Jose, CA, USA). Subsequently, blots were washed in TBS/0.05% Tween 20 and incubated for 1 h at room temperature in horseradish peroxidase-goat anti-rabbit or goat anti-mouse (Biorad) diluted 1/5000 in 5% skim milk in TBS/0.1% Tween 20. After additional washing, protein bands were detected by chemiluminescence using the Super Signal West Pico Substrate (ThermoFisher/Pierce). For both primary antibodies, the same membrane was re-probed for actin (HRP-conjugated rabbit anti-actin (#5125, 1/20,000) from Cell Signaling (Danvers, MA, USA)). Autoradiographs were scanned using a Biorad GS800 scanner. Band density was measured using the manufacturer’s software. Relative protein expression was normalized to actin band density. Each Western blot was repeated at least three times with independent protein samples.

### 2.7. Statistical Analysis

All of the experiments were performed at least three times and a minimum of three independent experiments were used for statistical analyses. The results were analyzed for statistically significant differences at the *p* < 0.05 level using the t-test or analysis of variance (ANOVA) test and Tukey’s post test for individual group means comparisons as appropriate. 

## 3. Results

**Figure 1:**
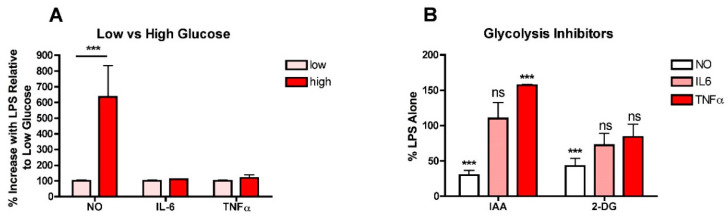
High glucose enhances NO production in LPS-treated BV2 microglial cells. (**A**) BV2 cells grown in low-glucose medium were treated with 25 ng/mL LPS for 24 h in either low-glucose (5 mM) or high-glucose (25 mM) medium. The cell culture supernatants were assayed for NO, IL6 and TNFα production. (**B**) BV2 cells grown in low-glucose medium were transferred to high-glucose medium in the absence or presence of 10 µM IAA or 10 mM 2-DG and treated with 25 ng/mL LPS for 24 h. The cell culture supernatants were assayed for NO, IL6 and TNFα production. The results are the average of a minimum of three independent experiments. *** *p* < 0.001 as compared to LPS alone. ns, not significant.

To determine whether high glucose enhances the production of NO and/or pro-inflammatory cytokines in LPS-stimulated microglia, BV2 microglial cells routinely grown in low- (5 mM) glucose medium were treated overnight with LPS in either low- or high- (25 mM) glucose medium and then the levels of nitrite as an index of NO production, and IL6 and TNFα in the culture supernatants were determined. As shown in Figure 1A, while high glucose significantly increased the production of NO, it had no effect on either IL6 or TNFα production. As a complement to these studies, BV2 cells routinely grown in low-glucose medium were transferred to high-glucose medium in the presence of iodoacetic acid (IAA), an inhibitor of GAPDH, a key enzyme in the glycolytic pathway, or 2-deoxyglucose (2-DG), a competitive inhibitor of phosphoglucoseisomerase, another key enzyme in glycolysis, and LPS was added. As shown in Figure 1B, while both IAA and 2DG were effective at preventing the LPS-mediated increase in NO production, neither had a significant effect on IL6 production and IAA actually increased TNFα production. Together, these studies suggested that the role of high glucose and increased glycolysis in exacerbating the pro-inflammatory phenotype in microglia was not as straightforward as previous studies would indicate.

**Figure 2:**
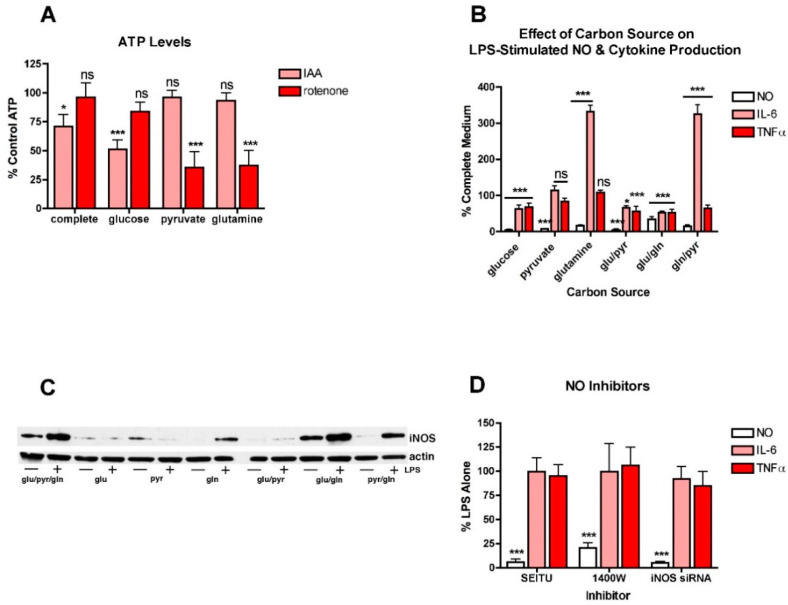
Different carbon sources have distinct effects on NO and cytokine production in LPS-treated BV2 cells. (**A**) BV2 cells were grown overnight in low-glucose medium and then transferred to medium containing only the indicated carbon source and either untreated or treated for 4 h with 10 µM IAA or rotenone. Cells extracts were prepared and assayed for ATP, the levels of which were normalized to the protein content of the extracts. The results are the average of a minimum of three independent experiments. * *p* < 0.05, *** *p* < 0.001 as compared to cells not treated with IAA or rotenone. ns, not significant. (**B**) BV2 cells were grown overnight in low-glucose medium and then transferred to medium containing the indicated carbon source(s) and then treated with 25 ng/mL LPS for 24 h. The cell culture supernatants were assayed for NO, IL6 and TNFα production. The results are the average of a minimum of three independent experiments. * *p* < 0.05, *** *p* < 0.001 as compared to medium containing glucose, pyruvate and glutamine. ns, not significant. (**C**) BV2 cells were grown overnight in low-glucose medium and then transferred to medium containing the indicated carbon source(s) and then left untreated or treated with 25 ng/mL LPS for 24 h. Cell extracts were prepared and equal amounts of protein analyzed by SDS-PAGE and Western blotting for iNOS and actin as a loading control. (**D**) BV2 cells were grown overnight in low-glucose medium and then transferred to high-glucose medium before treatment with 25 ng/mL LPS for 24 h in the absence or presence of 10 µM of the iNOS inhibitors SEITU or 1400W. As a complement to the inhibitors, cells were transfected overnight with iNOS siRNA. The next day, the cells were plated for experiments and grown overnight in low-glucose medium and then transferred to high-glucose medium before treatment with 25 ng/mL LPS for 24 h. In all cases, the cell culture supernatants were assayed for NO, IL6 and TNFα production. The results are the average of a minimum of three independent experiments. *** *p* < 0.001 as compared to LPS alone.

To further explore this idea, BV2 cells were treated with LPS in the presence of different carbon sources that favor either glycolysis or oxidative phosphorylation (OXPHOS). This approach was previously used to study the metabolism of cancer cells [26]. First, to show that the approach was effective at promoting either glycolysis or OXPHOS in the BV2 cells, they were transferred to the different carbon sources and then treated with either IAA, an inhibitor of glycolysis, or rotenone, an inhibitor of OXPHOS [26]. After 4 h, the cells were harvested and cell extracts assayed for ATP. As shown in Figure 2A, in BV2 cells grown in a carbon source that supports both glycolysis and OXPHOS (glucose, pyruvate and glutamine), neither IAA nor rotenone had a strong effect on ATP levels. However, in BV2 cells grown only in glucose, IAA but not rotenone significantly reduced ATP levels. In contrast, in BV2 cells grown in pyruvate or glutamine, carbon sources that favor OXPHOS, IAA had little or no effect while rotenone greatly reduced ATP levels. Thus, the different carbon sources are used for energy production by the BV2 cells as expected. 

The effects of the different carbon sources on NO and cytokine production were examined next. Surprisingly, none of the individual carbon sources promoted the production of NO (Figure 2B) or the induction of iNOS (Figure 2C) in response to LPS treatment nor did the combination of glucose and pyruvate. While the combinations of glucose and glutamine and glutamine and pyruvate stimulated some iNOS induction and NO production in response to LPS treatment, neither combination was as effective as the combination of all three carbon sources. In contrast, both IL6 and TNFα production were seen in the presence of each of the individual carbon sources. However, while TNFα production was either slightly reduced or unaffected by growth in a single carbon source, IL6 production was unaffected by growth in pyruvate alone but greatly enhanced by growth in glutamine alone. Furthermore, both pyruvate or especially glutamine alone were more effective at promoting IL6 production in the absence of glucose than in the presence, suggesting that glycolysis actually dampens LPS-induced IL6 production. Moreover, these results, along with the low- and high-glucose medium experiments, suggested that NO production and cytokine production can proceed independently of each other and are not necessarily regulated in tandem.

This idea was further investigated in studies with NO inhibitors. Two different NO inhibitors, SEITU and 1400W, were tested. As expected, both of these inhibitors dramatically decreased LPS-induced NO production but had little or no effect on cytokine production, even in the almost complete absence of NO (Figure 2D). Similar results were obtained using iNOS siRNA (Figure 2D).

**Figure 3:**
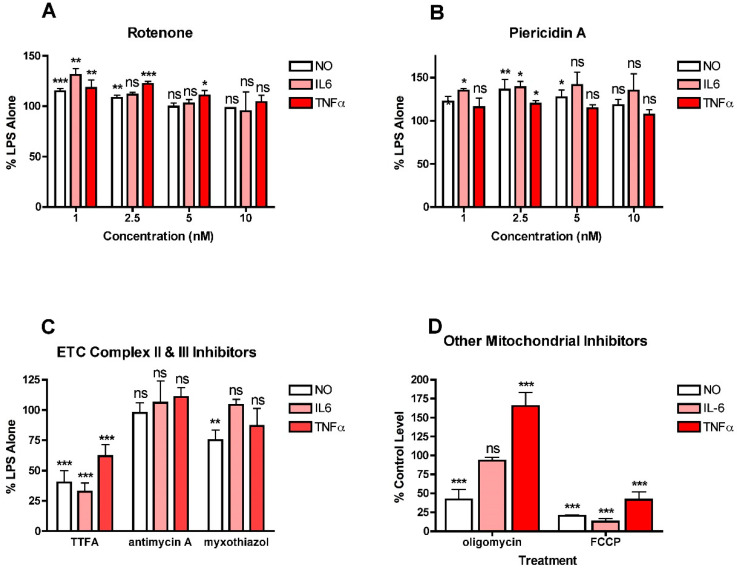
Effects of mitochondrial inhibitors on NO and cytokine production in LPS-treated BV2 cells. BV2 cells were grown overnight in low-glucose medium and then transferred to high-glucose medium before treatment with 25 ng/mL LPS for 24 h. (**A**) Cells were treated with LPS alone or in the presence of the indicated concentrations of the complex I inhibitor rotenone. (**B**) Cells were treated with LPS alone or in the presence of the indicated concentrations of the complex I inhibitor piericidin A. (**C**) Cells were treated with LPS alone or in the presence of 10 µM of the complex II inhibitor TTFA or 5 nM of the complex III inhibitor antimycin A or 2.5 nM of the complex III inhibitor myxothiazol. (**D**) Cells were treated with LPS alone or in the presence of 10 µM of the ATP synthase inhibitor oligomycin or 10 µM of the mitochondrial uncoupler FCCP. The cell culture supernatants were assayed for NO, IL6 and TNFα production. The results are the average of a minimum of three independent experiments. * *p* < 0.05, ** *p* < 0.01, *** *p* < 0.001 as compared to LPS alone. ns, not significant.

To further explore the role of mitochondria in NO and cytokine production by LPS-stimulated BV2 cells, a variety of electron transport chain (ETC) inhibitors were tested for their effects on NO, IL6 and TNFα production (Figure 3). For all of these studies, a wide range (1–10 µM) of inhibitor concentrations was tested initially and then further experiments focused on concentrations that did not induce significant toxicity. The effects of the inhibitors depended on the ETC complex that was targeted. Both of the complex I inhibitors, rotenone and piericidin A, at low doses, increased NO and cytokine production (Figure 3A,B). Interestingly, the effects were significant only at the lowest (low nanomolar) concentrations of the inhibitors tested. In contrast, the complex II inhibitor, TTFA, reduced the production of both NO and cytokines (Figure 3C) while the complex III inhibitors, antimycin and myxothiazol, had little effect on either NO or cytokine production (Figure 3C). The results with TTFA, antimycin and myxothiazol are only shown for the highest concentration tested that did not kill the cells. However, lower concentrations of antimycin A and myxothizaol also had no effects (not shown) and lower concentrations of TTFA were less effective (not shown). Interestingly, uncoupling mitochondria with FCCP greatly decreased NO and cytokine production (Figure 3D). However, while inhibiting ATP synthase with oligomycin reduced NO production by 50%, it had no effect on IL6 production and dramatically increased TNFα production (Figure 3D). Together, these results suggest that mitochondrial activity contributes to both NO and cytokine production in LPS-stimulated microglia but that the effects are highly dependent on the site of activity. 

**Figure 4:**
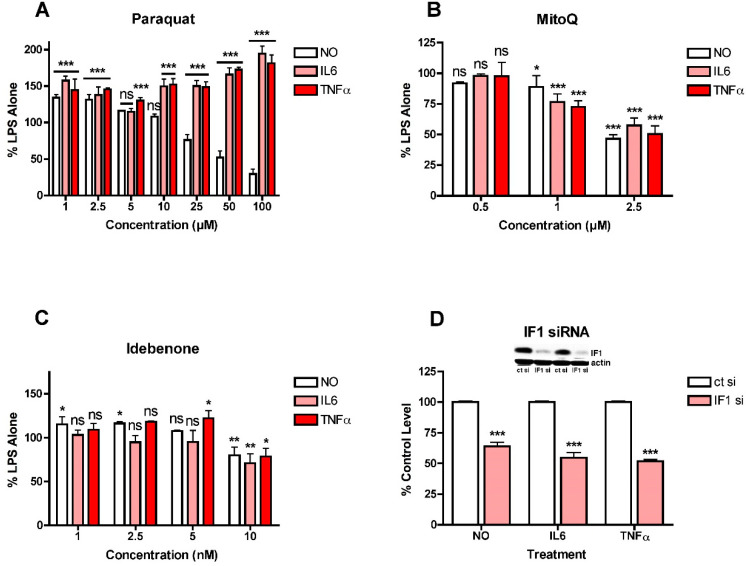
Effects of modulators of mitochondrial ROS levels on NO and cytokine production in LPS-treated BV2 cells. BV2 cells were grown overnight in low-glucose medium and then transferred to high-glucose medium before treatment with 25 ng/mL LPS for 24 h. (**A**) Cells were treated with LPS alone or in the presence of the indicated concentrations of paraquat. (**B**) Cells were treated with LPS alone or in the presence of the indicated concentrations of MitoQ. (**C**) Cells were treated with LPS alone or in the presence of the indicated concentrations of idebenone. (**D**) Cells were transfected overnight with control siRNA (ct si) or IF1 siRNA (IF1 si). Inset shows representative levels of IF1 knockdown. The next day, the cells were plated for experiments and grown overnight in low-glucose medium and then transferred to high-glucose medium before treatment with 25 ng/mL LPS for 24 h. The cell culture supernatants were assayed for NO, IL6 and TNFα production. The results are the average of a minimum of three independent experiments. * *p* < 0.05, ** *p* < 0.01, *** *p* < 0.001 as compared to LPS alone for (**A**–**C**) or ct si + LPS for (**D**). ns, not significant.

These observations suggested that mitochondrial ROS induction might play a role in the stimulation of NO and cytokine production by LPS in the BV2 cells since all three ETC complexes can give rise to ROS production [27], as can oligomycin treatment [28], while uncoupling the mitochondria with FCCP can reduce ROS production [27]. This idea was addressed using several different approaches. First, since complex I is known to be a source of mitochondrial ROS production and inhibition of this complex can enhance ROS production [27], it was asked how paraquat (PQ), a compound that stimulates ROS production from complex I by a different mechanism from rotenone and piericidin A [29], affects LPS-induced NO and cytokine production in the BV2 cells. As shown in Figure 4A, low concentrations of PQ enhanced LPS-induced NO and cytokine production while higher concentrations of PQ reduced NO production but further enhanced cytokine production. As a complement to these studies, the effects of the mitochondrially directed antioxidant MitoQ were also tested. As shown in Figure 4B, MitoQ had a similar dose-dependent, inhibitory effect on both NO and cytokine production but even the highest concentration shown (2.5 µM) only reduced the levels of all three by ~50%. Concentrations higher than 2.5 µM demonstrated significant toxicity. In addition, the coenzyme Q_10_ analogue and mitochondrial antioxidant idebenone [30] was also tested. It had a very modest effect on both NO and cytokine production resulting in ~25% reduction in the levels of all three but only at 10 µM, the highest concentration tested (Figure 4C). It has been argued that NADPH:quinone oxidoreductase (NQO1) is critical for the antioxidant activity of idebenone [30] so idebenone may be less effective in cells with low levels of this enzyme. However, the BV2 cells express high levels of NQO1 (not shown) so a lack of NQO1 is unlikely to be a factor in the modest effect of idebenone. As a complement to these studies, the effect of the knockdown of ATPase inhibitory factor 1 (IF1) on LPS-induced NO and cytokine production was examined since this physiological inhibitor of ATP synthase was shown to contribute to mitochondrial ROS production in cancer cells [31]. As shown in Figure 4D, loss of IF1 by ~85% reduced LPS-induced NO and cytokine production by ~40–50%, similar to results obtained with MitoQ. Together, the results shown in Figure 3 and Figure 4 suggested that different mitochondrial sources of ROS make distinct contributions to LPS-induced NO and cytokine production. They also further highlighted the disconnection between NO and cytokine production. In addition, since most only reduced cytokine production by 50% or less, they prompted an investigation into whether other sources of ROS also contribute to the pro-inflammatory phenotype and differentially affect LPS-stimulated NO and cytokine production.

**Figure 5:**
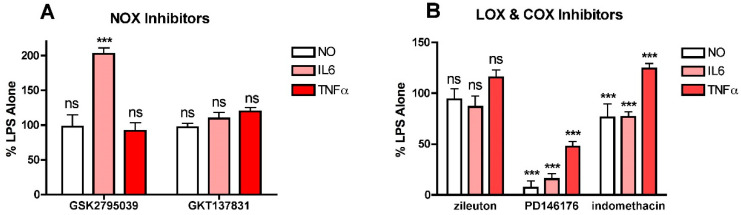
Effects of NOX, LOX and COX inhibitors on NO and cytokine production in LPS-treated BV2 cells. BV2 cells were grown overnight in low-glucose medium and then transferred to high-glucose medium before treatment with 25 ng/mL LPS for 24 h. Cells were treated with LPS alone or in the presence of (**A**) 10 µM of the NOX2 inhibitor GSK2795039 or the NOX1/4 inhibitor GKT137831; (**B**) 10 µM of the 5LOX inhibitor zileuton, the 15LOX inhibitor PD146176 or the COX inhibitor indomethacin. The cell culture supernatants were assayed for NO, IL6 and TNFα production. The results are the average of a minimum of three independent experiments. *** *p* < 0.001 as compared to LPS alone. ns, not significant.

To investigate this idea further, two other sources of cellular ROS production were examined to determine their effects on LPS-induced NO and cytokine production. Both NADPH oxidases (NOXs) and lipoxygenases (LOXs) promote ROS or lipid peroxide production, respectively, from various sites within cells and have been implicated in inflammation [5,32,33] so inhibitors of members of these families of enzymes were tested. For NOX inhibition, both the dual NOX1/4 inhibitor GKT137831 and the NOX2 inhibitor GSK2795039 were used since both NOX4 and NOX2 are known to be expressed in microglial cells [5] and NOX4 can be found in mitochondria [27]. For LOXs, both the ALOX15 inhibitor PD146176 and the ALOX5 inhibitor zileuton were tested since both of these LOXs are expressed by the BV2 cells (not shown). Neither the NOX1/4 inhibitor GKT137831 (Figure 5A) nor the ALOX5 inhibitor zileuton (Figure 5B) had any effect on either LPS-induced NO or cytokine production at concentrations up to 10 µM. In contrast, the ALOX15 inhibitor PD146176 was highly effective at lowering LPS-induced NO and IL6 production and also, but less robustly, TNFα production (Figure 5B). Surprisingly, the NOX2 inhibitor GSK2795039 had no effect on NO or TNFα production and significantly enhanced IL6 production (Figure 5A). The non-specific COX inhibitor indomethacin was also tested (Figure 5B). While it moderately reduced LPS-induced NO and IL6 production, it slightly enhanced TNFα production. These results suggest that one or more products of LOXs and specifically ALOX15 play an important role in promoting NO and cytokine production in response to LPS treatment. 

## 4. Discussion

One of the major observations from these studies is that mitochondrial activity is required for a full immune response in microglial cells. A number of studies have shown that the induction of inflammation in microglia cells by LPS or related molecules shifts microglial metabolism from mitochondrial OXPHOS to glycolysis [17,19,34], resulting in a decrease in mitochondrial ATP production. However, the data presented here using the different carbon sources show that a full immune response is only seen in the presence of carbon sources that can drive both glycolysis and OXPHOS. These results are supported by the anti-inflammatory effects of both the mitochondrial uncoupler FCCP and the complex II inhibitor TTFA. The results with FCCP are supported by the observation that in microglia cells lacking the mitochondrial uncoupling protein UCP2, the response to LPS is exacerbated [35]. In macrophages, succinate, the substrate of complex II, is increased in response to LPS treatment [36] although in this context the proinflammatory effects of succinate were associated with the stabilization of HIF1α and the induction of interleukin 1β but not IL6 or TNFα. Furthermore, succinate dehydrogenase, the complex II enzyme, was shown to play a key role in the pro-inflammatory phenotype in macrophages, at least with respect to interleukin 1β production [37]. In contrast, complex III inhibitors had no effect on LPS-induced NO or cytokine production in the BV2 cells, low doses of complex I inhibitors moderately enhanced both NO and cytokine production and, while the ATP synthase inhibitor oligomycin reduced NO production, it had no effect on IL6 production and increased TNFα production. Together, these observations suggest that specific signals arise from complex II activity that require a coupled mitochondrion and drive LPS-dependent inflammation. As discussed below, the results as well as the literature suggest that one of those signals could be mitochondrial ROS.

ROS have been implicated in the innate immune response in multiple studies (for reviews see [5,32,38]). There is evidence for several different sources for ROS including mitochondria, NOXs and LOXs. The results presented here indicate that in BV2 cells, the activity of mitochondria and potentially 15LOX are major contributors to the LPS-induced production of NO and cytokines. The results also suggest that there may be multiple sources of ROS within mitochondria which are all dependent on the membrane potential since TTFA, MitoQ and IF1 knockdown each only reduced NO and cytokine production by 40–50% while FCCP was much more effective. Indeed, the anti-inflammatory effects of FCCP are fully consistent with the dependence of mitochondrial ROS production on the protonmotive force [27]. Furthermore, mild mitochondrial uncoupling has been suggested as a therapeutic strategy for the regulation of mitochondrial ROS levels [27]. With regards to the 15LOX inhibitor PD146176, it should be noted that there is evidence that it can act not only as a 15LOX inhibitor but also as a radical trapping antioxidant [39]. However, the same study demonstrated a similar antioxidant activity for zileuton which was ineffective in the BV2 cell experiments.

As noted above, knockdown of IF1, a physiological inhibitor of ATP synthase [40], reduced the production of NO, IL6 and TNFα by a similar amount as MitoQ. IF1 overexpression promotes mitochondrial hyperpolarization and mitochondrial ROS production and this can be inhibited by MitoQ in tumor cells [31], suggesting that a major effect of MitoQ in the context of LPS-induced NO and cytokine production in the BV2 cells could be via a reduction in ROS generated through hyperpolarization of the mitochondrial membrane. This idea is consistent with the results with the mitochondrial uncoupler FCCP which also reduced both NO and cytokine production in response to LPS treatment in the BV2 cells although FCCP was more effective than either MitoQ or IF1 knockdown. However, it is not consistent with the results with the ATP synthase inhibitor oligomycin which, similar to IF1 overexpression, should increase the mitochondrial membrane potential but which in the LPS-treated BV2 cells reduced NO production and had no effect on IL6 production. In nerve cells, oligomycin was found to induce a huge increase in ROS production [28] and so the results with this compound in the BV2 cells may also reflect this difference between the effects of a modest (50% with IF1 overexpression [31]) and a massive (360% with oligomycin [28]) increase in ROS production.

Another important observation is that while LPS-induced NO, IL6 and TNFα production all appear to require some mitochondrial activity, IL6 seems to be particularly sensitive to the levels of glutamine in the culture medium. Glutamine has long been known to be important to the function of immune cells [41] but its precise role is still not clear. Glutamine is also required for the growth of some tumor cells and inhibition of glutamine metabolism has been investigated as a potential anti-cancer treatment [42]. Glutamine metabolism results in the production of several products that can contribute to energy production via non-canonical entry into the TCA cycle [42]. However, why that would specifically stimulate IL6 production is not obvious. Glutamine also stimulates activation of the mTOR pathway [42], which is known to contribute to the inflammatory response in microglial cells [43], but in this published study, rapamycin inhibited NO and TNFα production as well as IL6 production. Thus, although this is an exciting and novel observation, further elucidation of the mechanisms underlying this activity of glutamine are beyond the scope of this manuscript.

It is also important to note that these studies indicate that the LPS-induced production of NO, IL6 and TNFα can be uncoupled. First, the different carbon sources have distinct effects on NO, IL6 and TNFα production. Second, the various stimulators and inhibitors of glycolysis also have distinct effects on NO, IL6 and TNFα production as do some of the different mitochondrial and other enzyme inhibitors. Third, inhibitors of iNOS have little or no effect on either IL6 or TNFα production. Together, these observations has important implications for studies on neuroinflammation and anti-inflammatory compounds since it is clearly not sufficient to look at only one or even two readouts of inflammation to determine whether a compound has truly broad based pro- or anti-inflammatory effects. 

This study also has several limitations. First, the effects of most of the treatments on cellular metabolism was not assessed. Thus, while it is assumed that the various treatments did produce the expected changes in cellular metabolism, this was not confirmed by direct testing. Second, no studies were performed to directly tie the effects of the various treatments to neuronal dysfunction or AD. Nevertheless, the work presented in this paper provides the basis for further studies to address these questions. 

## 5. Conclusions

These studies were initiated to explore the idea that diabetes-related hyperglycemia could play a role in the exacerbation of age-related chronic neuroinflammation and thereby contribute to the development and/or progression of AD. However, while the results indicate that high glucose contributes to the LPS-induced production of NO, it does not seem to further exacerbate the production of either IL6 or TNFα under the same conditions. In contrast, mitochondrial dysfunction, which is also implicated in both diabetes and AD [44], and specifically inhibition of complex I, does contribute to the enhanced production of cytokines as well as NO under pro-inflammatory conditions. Thus, these results suggest a two-step mechanism whereby age-dependent activation of microglia is further enhanced by stresses related to diabetes as well as potentially other insults. Importantly, the results also suggest that the pro-inflammatory effect of mitochondrial dysfunction could also contribute to the exacerbation of neuroinflammation in the context of Parkinson’s disease (PD) since both rotenone and PQ significantly enhanced markers of neuroinflammation at low nanomolar concentrations and both toxins are associated with PD [45]. Furthermore, consistent with what is shown in this study, there is evidence that low doses of rotenone and LPS act synergistically to activate microglia and induce neurodegeneration in mixed primary mesencephalic neuron–glia cultures [46]. Thus, although the results do not support the original hypothesis that high glucose exacerbates the activation of microglial cells by a pro-inflammatory stimulus by enhancing glycolysis, instead they strongly suggest that alterations in mitochondrial function synergize with inflammatory stimuli to produce a more robust pro-inflammatory response. This observation could have important implications for the treatment of age-related neurodegenerative diseases. 

## Data Availability

The data is available upon request.
